# Pathophysiology and Treatment of Heart Failure with Preserved Ejection Fraction: State of the Art and Prospects for the Future

**DOI:** 10.36660/abc.20190111

**Published:** 2020-01

**Authors:** Sara Lopes Fernandes, Rita Ribeiro Carvalho, Luís Graça Santos, Fernando Montenegro Sá, Catarina Ruivo, Sofia Lázaro Mendes, Hélia Martins, João Araujo Morais

**Affiliations:** 1 Centro Hospitalar de Leiria, Leiria - Portugal

**Keywords:** Heart Failure/physiopathology, Heart Failure/diagnosis, Heart Failure/drug therapy, Systolic Volume, Heart Failure/complications

## Introduction

Heart failure (HF) is extremely prevalent and has a considerable impact on mortality and quality of life.^1^ It affects nearly 1-3% of the adult population in developed countries, exponentially increasing with age and affecting more than 10% of the population over 70 years. Given the increase in the average life expectancy, better diagnostic methods and increased comorbidities, a greater prevalence of heart failure is expected.^2^

It is a clinical syndrome characterized by classic symptoms (such as fatigue, dyspnea) that may be accompanied by clinical signs (elevated jugular pressure, pulmonary crackles and peripheral edema). It is caused by structural and/or functional cardiac abnormalities, resulting in reduced cardiac output and/or elevated intracardiac pressures at rest or during stress.^3^

The main terminology used to describe HF is based on the measurement of the left ventricular ejection fraction (EF), differentiating patients with reduced <40% (HFrEF), mid-range 40-49% (HFmrEF) and preserved ≥50% (HFpEF) ejection fraction. This classification is important due to different underlying etiologies, pathophysiology, available treatment and its respective response.^3^ HFpEF accounts for about half of the cases of HF in developed countries.^4^

Its pathophysiology is complex, heterogeneous and still poorly understood. The wide variety of phenotypes resulting from the several pathophysiological mechanisms, comorbidities and dominant clinical characteristics, make diagnosis and treatment extremely challenging.^4^

Unlike HFrEF, no treatment has unquestionably shown a reduction of morbidity or mortality in patients with HFpEF or HFmrEF. Several clinical trials evaluating drugs proven to be effective in HFrEF have failed to demonstrate prognostic benefits in these patients.^4^ Current recommendations are based on symptom relief, screening, and treatment of comorbidities.^3^

New therapies are presently under research, especially directed at the pathophysiological mechanisms of the disease.^5^ This review addresses the pathophysiology of HFpEF and summarizes the studies that have been carried out regarding treatment, including failures, hopes and future prospects.

For this article, we carried out a systematic search in three databases: Medline - Pubmed, ISI Web of knowledge and Scopus, using the following keywords in English and Portuguese: "Heart failure AND preserved ejection fraction", "Heart failure AND preserved ejection fraction AND physiopathology” and “Heart failure AND preserved ejection fraction AND treatment". The study was conducted between January and March of 2019. Prospective and retrospective studies were included. Clinical cases, abstracts presented at conferences (not published as articles) and studies with a sample size of less than 10 patients were excluded. The eligibility of each study was independently assessed by three researchers. The divergent opinions regarding the relevance of the articles were resolved by consensus among the authors.

### Pathophysiology

The pathophysiology of the disease is complex and remains insufficiently understood. It is known that these patients are generally older, females and have multiple cardiovascular comorbidities, such as hypertension, atrial fibrillation (AF), coronary artery disease (CAD), pulmonary hypertension (PH), and non-cardiovascular diseases such as diabetes, chronic kidney disease (CKD), anemia, chronic obstructive pulmonary disease (COPD), among others. They also have a higher percentage of non-cardiovascular pathologies, with a great impact on morbidity and mortality, and a lower incidence of acute myocardial infarction (AMI) as well as sudden cardiac death or death from HF.^6^

**Figure 1:**
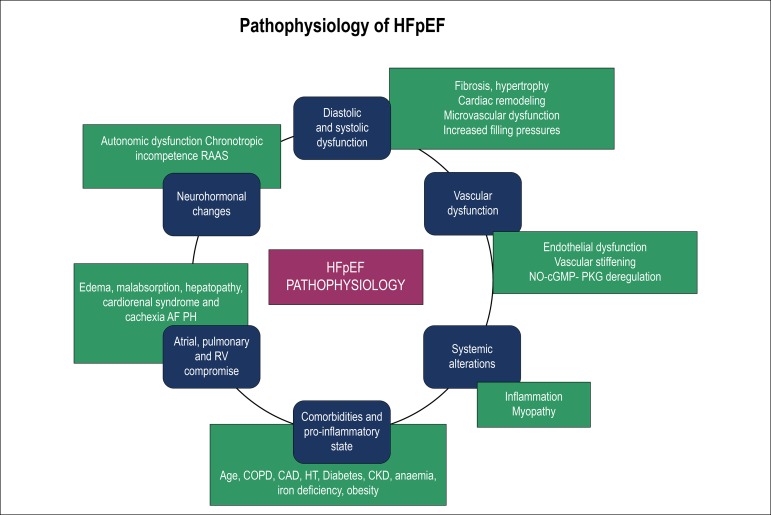
Pathophysiology of HFpEF - possible mechanisms involved. AF: atrial fibrillation; CAD: coronary artery disease; CKD: chronic kidney disease; COPD: chronic obstructive pulmonary disease; HFpEF: heart failure with preserved ejection fraction; HT: arterial hypertension; NO-cGMP-PKG: nitric oxide, reduced cyclic guanosine monophosphate and protein kinase G; PH: pulmonary hypertension; RAAS: renin-angiotensin-aldosterone system; RV: right ventricle.

Historically, HFpEF was exclusively associated with diastolic dysfunction, opposed to HFrEF, which was associated with systolic dysfunction. It is currently known that this is not such a clear-cut matter, as both types of HF may also show systolic and/or diastolic dysfunction. Different mechanisms are involved in HFpEF. This is thought to result from a complex variety of cardiac, vascular and systemic dysfunctions, with the contribution of several comorbidities.^4^ (Figure 1)

Diastolic dysfunction is usually present and results from structural changes (cardiac fibrosis, hypertrophy and remodeling ), microvascular dysfunction and metabolic abnormalities, with increased stiffness and decreased cardiac compliance. This leads not only to an increase in LV filling pressures, but also to structural and functional changes at the atrial, pulmonary and right ventricular levels, due to a rise in upstream pressures. The systolic reserve is also affected, mainly due to changes in the ventricular-vascular coupling ratio.^4^

Atrial changes, with dilatation and remodeling, favor the appearance of AF. Pulmonary hypertension, present in 53-83% of the cases and associated with a worse prognosis, also seems to contribute to the disease progression.^7^ The onset of right ventricular dysfunction, with systemic venous congestion, also predicts worse results, associated with edema, malabsorption, congestive hepatopathy, cardiorenal syndrome and cachexia.

Another mechanism involved is chronotropic incompetence, with inadequate heart rate (HR) variations, probably due to autonomic nervous system dysfunctions.^4^ Electrical and/or mechanical, systolic and diastolic asynchronies were also observed in some patients.^7^ Its magnitude is related to the extent of diastolic dysfunction and exercise capacity.^4^

Many of these changes are not apparent, nor do they entail any impairment at rest, with functional reserve limitations being evident only under stress.

Neurohormonal alterations, such as autonomic dysfunction and activation of the renin-angiotensin-aldosterone system (RAAS) are also important mechanisms involved.^4^

At the vascular level, we can observe endothelial dysfunction, systemic inflammation, increased vessel stiffness and impaired vasodilation. A potential mechanism could be the deregulation of the NO-sGC-cGMP-PKG signaling pathway (nitric oxide, soluble guanylate cyclase, reduced cyclic guanosine monophosphate and protein kinase G), which is responsible for smooth muscle relaxation, cardiac protection, gene transcription, endothelial permeability and platelet inhibition.^5^ At the peripheral level, musculoskeletal changes seem to contribute to aerobic capacity reduction, with less exercise tolerance.

Both the age and the several comorbidities intensify these mechanisms and contribute to disease progression. The interaction between the various pathophysiological factors and comorbidities and the relative dominance of each of them makes this pathology complex and heterogeneous, making its diagnosis and treatment extremely difficult. A subgroup analysis with certain phenotypes can facilitate this process by allowing a more particular and direct approach.^4^

### Diagnosis

The diagnosis of HFpEF is more challenging than the diagnosis of HFrEF. There have been several proposed classifications and inclusion criteria in the conducted studies, contributing to the enormous heterogeneity of patients assessed in the clinical trials.^1^

The current guidelines proposed by the European Society of Cardiology suggest the existence of 3 diagnostic criteria: symptoms and signs of HF, LVEF ≥ 50%, elevated levels of natriuretic peptides and relevant structural heart disease and/or diastolic dysfunction.^3^ Notwithstanding these recommendations, and given the clinical heterogeneity, absence of pathognomonic criteria and the multiplicity of differential diagnoses, there are several challenges and uncertainties to be faced.^5^

### Treatment

Unlike HFrEF, no treatment has yet shown a reduction in morbidity or mortality. Therefore, current recommendations are based on symptom relief with diuretics, screening and treatment of comorbidities.^3^ Diuretics are recommended in case of congestion, for symptom relief, regardless of the LVEF.^3^ They are widely used, especially loop diuretics, even though there are no specific recommendations concerning which diuretic therapy should be followed.^8^

Several clinical trials have studied the effect of drugs proven to be effective in HFrEF for the treatment of patients with HFpEF (Table 1-A).

**Table 1:** A) Main studies performed in patients with HFpEF using effective drugs in the treatment of the HFrEF; B) New drugs and new approaches in HFpEF

**A**	**Clinical Trial**	**Year**	**Intervention**	**Patients, n**	**Major inclusion criteria**	**Mean follow-up**	**Main conclusions**
Beta Blockers	SENIORS^9^	2005	Nebivolol vs. placebo	2128	≥70 years, mean LVEF of 36%, 35% with LVEF > 35%, 68% CAD	1,8 years	Well tolerated and effective in reducing mortality and CV hospitalization (HR 0.86, 95%CI: 0.74-0.99; p = 0.039)
ACEI/ARB	CHARM Preserved^13^	2003	Candesartan vs. placebo	3023	>18 years, LVEF > 40%, NYHA II-IV	3 years	Tends towards a reduction in CV mortality and HF hospitalization (*unadjusted* HR 0.89 95%CI: 0.77-1.03, p = 0.118; *adjusted* 0.86 [0.74-1·0], p = 0.051)
	PEP-CHF^14^	2006	Perindopril vs. placebo	850	≥70 years, HF under diuretic therapy, diastolic dysfunction, without systolic or valvular dysfunction	2,1 years	No difference in mortality or CV hospitalization (HR 0.92 95%CI: 0.70-1.21, p = 0.545). Some improvements in symptoms, exercise capacity and HF hospitalization in the first year of follow-up (younger patients with AMI or hypertension)
	I-PRESERVE^12^	2008	Irbesartan vs. placebo	4128	>60 years, LVEF > 45%, NYHA II-IV	4.1 years	No difference in mortality or CV hospitalization (HR 95%CI: 0.86-1.05, p = 0.35)
	Enalapril^15^	2010	Enalapril vs. placebo	71	70 ± 1 years (80% women), LVEF ≥ 50%, Compensated HF and controlled Hypertension	1 year	No impact on exercise capacity (p = 0.99), aortic distensibility (p = 0.93), ventricular volume and mass (p = 1) or quality of life (p = 0.07)
MRA	Aldo -DHF^16^	2013	Spironolactone vs. placebo	422	≥50 years, LVEF ≥ 50%, NYHA II-III, diastolic dysfunction	1 year	Improved diastolic function (E/e' p < 0.001, ventricular remodeling p = 0.09 and neurohormonal activation; p = 0.03). Did not improve exercise capacity, symptoms or quality of life (p = 0.03)
	TOPCAT^17^	2014	Spironolactone vs. placebo	3445	≥50 years, LVEF ≥ 45%, Symptomatic HF, hospitalization within last 12 months or elevated natriuretic peptides	3.3 years	No reduction in CV mortality, cardiac arrest or HF hospitalization (HR 0.89, 95%CI: 0.77-1.04, p = 0.14). Some benefit in terms of natriuretic peptide levels
ARNI	PARAMOUNT^19^	2012	Sacubitril/valsartan vs. valsartan	301	LVEF ≥ 45%, NYHA II-III and NT-proBNP > 400 pg/ml	12 and 36 weeks	Reduction in NT-proBNP at 12 weeks (HR 0.77, 95%CI: 0.64-0.92, p = 0.005); LA volume reduction (p = 0.003) and NYHA class improvement (p = 0.05) at 36 weeks
	PARAGON^20^	2019*	Sacubitril/valsartan vs. valsartan	4300	LVEF ≥ 45%, NYHA II-IV, elevated natriuretic peptides and evidence of structural heart disease	>2 years	Evaluation of CV mortality and HF hospitalizations
Ivabradine	If- Channel Inhibitors^22^	2013	Ivabradine vs. placebo	61	LVEF ≥ 50%, diastolic dysfunction, NYHA II-III, sinus rhythm, HR ≥ 60 bpm, exercise capacity <80% for age and gender	7 days	Increased exercise capacity (p = 0.001), with improvement in hemodynamic status during the exercise (p = 0.004); improved LV filling pressure (p = 0.02)
	EDIFY^21^	2017	Ivabradine vs. placebo	179	LVEF ≥ 45%, NYHA II-III, sinus rhythm, HR ≥70 bpm, NT-proBNP ≥ 220 pg/mL (BNP ≥ 80 pg/mL	8 months	No improvement in diastolic function (HR 1.4 90%CI: 0.3-2.5, p = 0.135), exercise capacity (p = 0.882) or NT-proBNP level (HR 1.01, 90%CI: -0.86-1.19; p = 0.882)
Digoxin	DIG PEF^23^	2006	Digoxin vs. placebo	988	LVEF > 40% (mean 53%), sinus rhythm	3.1 years	No effect on natural history endpoints such as mortality and hospitalizations (HR 0.82; 95%CI: 0.63-1.07; p = 0.136)
Nitrates and Nitrites	NEAT HFpEF^24^	2015	Isosorbide mononitrate vs. placebo	110	≥50 years, LVEF ≥ 50%, evidence of HF	6 weeks	No effect on quality of life (p = 0.37) or NT-proBNP levels (p = 0.22); Reduction in daily activity level (-381 95%CI -780-17, p = 0.06) and increased symptoms of HF
	Inorganic nitrate on exercise capacity^25^	2015	NO3-rich beetroot juice vs. placebo (single dose)	17	Symptomatic HF, LVEF > 50%	12 days	Increased exercise capacity (p = 0.04) (reduction in systemic vascular resistance, increased cardiac output and increased oxygen delivery)
Sildenafil	RELAX^26^	2013	Sildenafil vs. placebo	216	LVEF ≥ 50%, NYHA II-IV, NT-proBNP > 400 pg/mL, Peak VO_2_ < 60%, or elevated LV filling pressures	24 weeks	No effect on exercise capacity (p = 0.90), clinical status (p = 0.85) or diastolic function (p = 0.16). Worsening of renal function, NTproBNP, endothelin-1 and uric acid
sCG Stimulators	DILATE-1^27^	2014	Riociguat vs. placebo (single dose)	39	≥18 years, LVEF > 50% and PH; mPAP ≥ 25 mmHg and PCWP > 15 mmHg	30 days	Well tolerated; improved exploratory hemodynamic and echocardiographic parameters; No impact on mPAP (p = 0.60)
	SOCRATES-Preserved^28^	2016	Vericiguat vs. placebo	470	LVEF ≥ 45%, NYHA II-IV, elevated natriuretic peptides	12 weeks	No effect on NT-proBNP (p = 0.20) or LA volume (p = 0.37). Some potential in improving quality of life (p = 0.016), particularly with higher doses
Ranolazine	RALI-DHF^29^	2013	Ranolazine vs. placebo	20	LVEF ≥ 45%, E/E` > 15 or NT-proBNP > 220pg/mL, tau ≥ 50ms, LVEDP ≥ 18 mmHg	14 days	Despite hemodynamic improvements at 24 h, there was no effect on diastolic function parameters
**B**	**Clinical Trial**	**Year**	**Intervention**	**Patients, n**	**Major inclusion criteria**	**Mean follow-up**	**Main conclusions**
Albuterol	BEAT - HFpEF^30^	2019	Albuterol vs. placebo	30	LVEF ≥ 50%, elevated LV filling pressures, PCWP > 15 mmHg and/or ≥ 25 mmHg during exercise	-	Symptom evaluation through its effect on pulmonary vascular resistance at rest and during exercise
Shunt	REDUCE LAP-HF I^31^	2017	Interatrial septal shunt device vs. sham procedure	94	LVEF>40% and elevated PCWP	1 month	Showed to be safe and effective; Reduction of PCWP (p = 0.028) without significant increase in PAP or pulmonary vascular resistance
Monitoring	CHAMPION^34^	2014	Hemodynamic monitoring vs. control	119	LVEF > 40% (mean 50.6%), NYHA III	17.6 months	Significant reduction in HF hospitalizations (HR 0.50; 95%CI: 0.35-0.70; P < 0.0001)
Exercise	EX DHF^36^	2011	Supervised resistance training vs. usual care	64	> 45 years, LVEF ≥ 50%, NYHA II-III, diastolic dysfunction, sinus rhythm and ≥ 1 CV risk factor	3 months	It showed to be achievable, safe and effective; Improved functional capacity, diastolic function and quality of life (´p < 0.001)
Comorbidities	OPTIMIZE-HFPEF^38^	2016	Systematic screening and optimal treatment of comorbidities vs. usual care	360	>60 years, LVEF ≥ 50%, NYHA II-IV	2 years	Assessment of clinical status
Pacing	RAPID-HF^39^ (NCT02145351)	2019	Dual chamber pacemaker with pacing on vs. pacing off	30*	LVEF ≥ 50%, NYHA II-III, diastolic dysfunction and chronotropic incompetence	4 weeks	Assessment of exercise capacity, symptoms and quality of life
Iron Supplementation	FAIR^40^ (NCT03074591)	2019	Ferric Carboxymaltose IV vs placebo	200*	LVEF ≥ 45%, NYHA II-III, diastolic dysfunction, iron deficiency, Hb 9-14g/dL	52 weeks	Evaluation of exercise capacity, quality of life, NYHA functional class, mortality and HF hospitalizations
SGLT2 Inhibitors	EMPERIAL Preserved^46^ (NCT03448406)	2019	Empagliflozin vs. placebo	300*	LVEF > 40%, NYHA II-IV, NT-proBNP > 300pg/mL, 6 min-walking distance ≤ 350 metros	12 weeks	Assessment of exercise capacity measured by the 6 min-walking distance
	Preserved-HF^47^ (NCT03030235)	2019	Dapagliflozin vs. placebo	320*	LVEF ≥ 45%, NYHA II-III, NT-proBNP ≥ 225pg/mL or BNP ≥ 75 pg/mL	12 weeks	NT-proBNP evaluation
	EMPEROR-Preserved^48^ (NCT03057951)	2021	Empagliflozin vs. placebo	6000*	LVEF > 40%, NYHA II-IV, NT-proBNP > 300pg/mL	38 months	Evaluation of CV death and HF hospitalization

AMI: acute myocardial infarction; CAD: coronary artery disease; CO: cardiac output; CV: cardiovascular; HF: heart failure; HR: hazard ratio; LA: left atrium; LVEF: left ventricle ejection fraction; mPAP: mean pulmonary artery pressure; NYHA: New York Heart Association; PAP: pulmonary artery pressure; PCWP: Pulmonary Capillary Wedge Pressure; 95% CI: 95% confidence interval;

* Estimated target number.

#### 1. Beta-blockers (BB)

The *Randomized trial to determine the effect of nebivolol on mortality and cardiovascular hospital admission in elderly patients with heart failure,* the “SENIORS” trial,^9^ evaluated the effect of nebivolol in patients over 70 years with a history of HFrFE and HFpEF (LVEF > 35%). Despite the reduction in morbidity and mortality, most patients had reduced LVEF (mean 36%) and a history of coronary artery disease and, thus, it was not possible to extrapolate the results to patients with true HFpEF. In a meta-analysis performed later, the BB were the only drugs able to reduce cardiovascular and all-cause mortality.^10^ However, patients with different LVEF were included, so the obtained results might possibly have been due to pleiotropic effects in patients with HFmrEF. Recently, our group showed the role of BB in patients with acute coronary syndrome and HFmrEF, demonstrating a reduction of in-hospital mortality, as well as myocardial revascularization.^11^

#### 2. Angiotensin-converting enzyme inhibitor (ACEI)/Angiotensin receptor blocker (ARB)

In spite of the proven efficacy in patients with HFrFE, post-AMI, hypertension and/or high cardiovascular risk, the benefit in patients with HFpEF is limited.^12^ The *Effects of candesartan in patients with chronic heart failure and preserved left-ventricular ejection fraction,* the *“*CHARM-Preserved*”* trial,^13^ showed that candesartan, despite reducing hospital admissions, had no impact on cardiovascular mortality when compared to placebo. *The perindopril in elderly people with chronic heart failure,* the “PEP-CHF” trial^14^ evaluated the impact of perindopril in patients with diastolic HF, showing no statistical benefit on long-term mortality or hospitalization. However, it appeared to improve symptoms, exercise capacity and HF hospitalization, particularly in younger patients with a history of AMI or hypertension. In addition, irbesartan showed no benefits in terms of mortality, hospitalizations or quality of life assessed in the *Irbesartan in Patients with Heart Failure and Preserved Ejection Fraction,* the “I-PRESERVE” trial.^12^ Another clinical trial showed that 12 months of enalapril had no effect on exercise capacity, aortic distensibility, ventricular parameters or quality of life.^15^

#### 3. Mineralocorticoid/aldosterone receptor antagonists (MRA)

Activation of the mineralocorticoid receptors contributes to the pathophysiology of HF through sodium and water retention, potassium loss, endothelial dysfunction, inflammation, fibrosis, and hypertrophy.^16^ These patients would be expected to benefit from MRA use. The *Effect of Spironolactone on Diastolic Function and Exercise Capacity in Patients With Heart Failure With Preserved Ejection Fraction,* the “ALDO-DHF” trial,^16^ showed advantages in structural reverse cardiac remodeling and improved diastolic function, but did not affect maximal exercise capacity, patient symptoms, or quality of life. The study did not have enough power to evaluate the effect of spironolactone on HF hospitalizations or mortality. The *Spironolactone for Heart Failure with Preserved Ejection Fraction,* the “TOPCAT” trial,^17^ added more information and assessed the clinical impact of spironolactone on HFpEF. Although it did not significantly reduce the primary outcome (cardiovascular death, cardiac arrest or HF hospitalization), a subgroup analysis revealed benefits in patients with elevated natriuretic peptide levels. These results have led current American guidelines to consider spironolactone in selected groups of patients with symptomatic HFpFE, particularly those with high natriuretic peptide levels, aiming to reduce hospitalizations (Class IIb).^18^

#### 4. Angiotensin receptor neprilysin inhibitor (ARNI)

Increasing natriuretic peptide levels with ARNI is expected to improve myocardial relaxation, natriuresis, vasodilation and attenuation of sympathetic and fibrotic activity, aiming to improve cardiac function and symptoms. *The angiotensin receptor neprilysin inhibitor LCZ696 in heart failure with preserved ejection fraction*”, the PARAMOUT”^19^ trial: a phase II study, randomized 301 patients with HFpEF to receive either ARNI or valsartan. The primary endpoint, which was the change in NT-proBNP levels at 12 weeks, was significantly better in the sacubitril/valsartan group. At 36 weeks, there was also a reduction in left atrial (LA) volume, a marker of LV filling pressures, and an improvement in the NYHA functional class. *Angiotensin Receptor Neprilysin Inhibition in Heart Failure With Preserved Ejection Fraction, the* “PARAGON”^(20 )^trial: a phase III study, will assess the clinical benefit and safety of this drug in chronic symptomatic patients with HFpEF.

#### 5. Ivabradine

An elevated heart rate (HR) is a predictive factor of worse outcomes and increased mortality in patients with heart failure, including those with HFpEF. Ivabradine is a specific and selective inhibitor of the sinoatrial node, *if current*, and thereby decreases HR in patients with sinus rhythm.^21^ In patients with HFpEF, short-term treatment increased exercise capacity by improving LV filling pressures.^22^ As these patients are mostly symptomatic during exercise, therapies targeting hemodynamic changes during exercise may be useful. The *Effect of ivabradine in patients with heart failure with preserved ejection fraction,* the “EDIFY” trial,^21^ evaluated the effect of the drug over 8 months. Unlike the previous study, there was no improvement in the evaluated parameters (diastolic function, exercise capacity and NT-proBNP reduction). Future studies may show benefits in certain subgroups.

#### 6. Digoxin

Digoxin is also part of the therapeutic algorithm in HFrEF, although it is not the first-line therapy.^3^ A potential benefit in patients with diastolic dysfunction and HFpEF could arise from its neurohormonal action. The *Effects of Digoxin on Morbidity and Mortality in Diastolic Heart Failure,* the “DIG PEP” trial,^23^ showed no effect on the natural history endpoints, such as mortality and hospitalizations. Although it was associated with a trend toward reduction in HF hospitalizations, it did not affect the overall results, partly because of a non-significant increase in the risk of hospitalization for unstable angina.

#### 7. Nitrates and Nitrites

Another pathophysiological mechanism involved in HFpEF is the deregulation of the NO-sGC-cGMP-PKG pathway. A possible therapeutic approach would consist in the use of drugs that act at this level, such as nitrates, phosphodiesterase-5 inhibitors, riociguat and vericiguat.

The *Isosorbide Mononitrate in Heart Failure with Preserved Ejection Fraction,* the “NEAT- HFpEF” trial,^24^ evaluated an isosorbide mononitrate regimen, using increasing doses, for 6 weeks. In addition to the lack of improvement in quality of life or NT-proBNP levels, there was a reduction in daily activity level and increased HF symptoms. Other mechanisms eventually limit the hemodynamic benefits of organic nitrates and predispose patients to excessive hypotension and other adverse effects.

The hypothesis that the results would be better with inorganic nitrates (NO3) was tested in a pilot study that assessed exercise capacity and the impact on vasculature and skeletal muscle, using NO3-rich beetroot juice. Although the primary endpoint was not reached, the results seemed to be positive.^25^ It will be important to confirm the results in larger, long-term trials.

#### 8. Sildenafil

Inhibition of phosphodiesterase-5 seems to reverse cardiac remodeling and improve vascular, neuroendocrine and renal function, with clinical improvement in patients with idiopathic pulmonary arterial hypertension (PAH) and HFrEF. The *Effect of Phosphodiesterase-5 Inhibition on Exercise Capacity and Clinical Status in Heart Failure With Preserved Ejection Fraction,* the “RELAX” trial,^26^ evaluated these parameters in patients with HFpEF, comparing sildenafil with placebo for 24 weeks. Not only was there no improvement in exercise capacity, clinical status, cardiac remodeling or diastolic function, but also the renal function and NTproBNP, endothelin-1 and uric acid levels were adversely affected. In the subgroup of patients with HFpEF and severe pulmonary vascular disease, the results might perhaps be different and more encouraging.^5^

#### 9. sCG Stimulators (Riociguat and Vericiguat)

Pulmonary hypertension (PH) is frequently seen in patients with HF and has been shown to be a major determinant of worse outcomes, thereby representing a potential novel therapeutic target in HFpEP. Riociguat is a novel soluble guanylate cyclase (sGC) stimulator. Its vasodilatory, antifibrotic, antiproliferative and antiinflammatory effect has shown to be efficient in pulmonary arterial hypertension and chronic thromboembolic PH with LV systolic dysfunction. The *Acute Hemodynamic Effects of Riociguat in Patients With Pulmonary Hypertension Associated With Diastolic Heart Failure,* the “DILATE-1” trial,^27^ evaluated its effect in patients with PH and diastolic dysfunction. It was an initial study, which assessed a small number of patients and used single doses of riociguat. Despite being well tolerated and improving exploratory hemodynamic and echocardiographic parameters, further studies with larger sample sizes and longer duration are needed to assess its long-term clinical effect.

In the *Vericiguat in patients with worsening chronic heart failure and preserved ejection fraction,* the “SOCRATES-Preserved” trial,^28^ 12 weeks of treatment with vericiguat also did not change the primary endpoints, NT-proBNP levels and LA volume. Some potential to improve quality of life has been suggested, particularly at higher doses, which may be tested in further studies, possibly with higher doses, longer follow-up and additional endpoints.

#### 10. Ranolazine

It is known that both HF and ischemic heart disease show increased late sodium current on intracellular calcium cycling, compromising cardiac relaxation. By inhibiting the late sodium channels with ranolazine, an improvement in the diastolic function would theoretically be expected.^6^ The *RAnoLazIne for the Treatment of Diastolic Heart Failure in Patients With Preserved Ejection Fraction, the “*RALI-DHF” trial^29^ was an exploratory study that evaluated the drug in patients with HFpEF. Despite hemodynamic improvements after 24 hours, there were no significant changes in diastolic function after 14 days of treatment.

#### Another Direction

**Figure 2:**
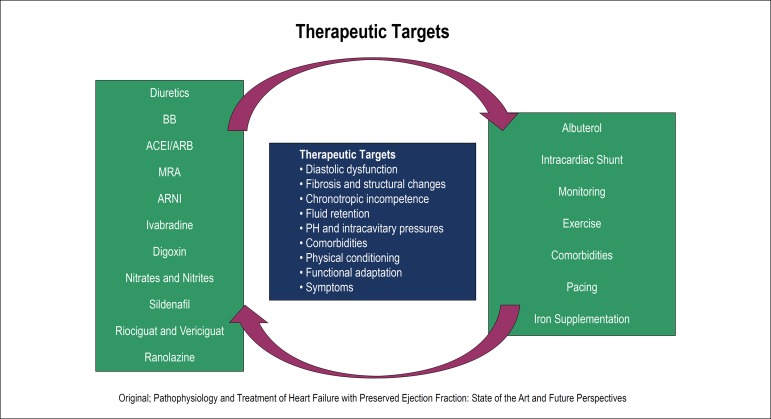
Potential therapeutic targets and drugs evaluated in HFpEF. ACEI/ARB: angiotensin-converting enzyme inhibitors/angiotensin II receptor blocker; ARNI: angiotensin receptor neprilysin inhibitor; BB: Beta Blockers; MRA: mineralocorticoid receptor antagonists; PH: pulmonary hypertension.

The failure of clinical trials in testing proven effective drugs in HFrEF, has led to a new direction in the treatment of patients with HFpEF. Attempts were made to better understand the pathophysiological mechanism of the disease and act on those different pathways (Figure 2). New drugs have been tested and new approaches are under research. (Table 1-B)

#### 11. Albuterol

Given the frequent lung involvement in these patients, drugs acting at this level are being tested. This is the case of albuterol, an inhaled bronchodilator. The *Inhaled Beta-adrenergic Agonists to Treat Pulmonary Vascular Disease in Heart Failure With Preserved EF,* the *“*BEAT - HFpEF” trial,^30^ aims to assess the impact of this drug on symptoms, through its effect on pulmonary vascular resistance at rest and during exercise.

#### 12. Interatrial septal shunt

It is known that the atrial volume and pressure overload not only contributes to the development of symptoms and exercise intolerance, but it is also a major determinant of morbimortality. Pulmonary capillary wedge pressure (PCWP) is an invasive hemodynamic parameter with prognostic value, which reflects the pressure in the LA and pulmonary veins. Based on these hemodynamic changes and in view of the limited success of pharmacological management of patients with HFpEF, an interatrial communication device was developed, which is used to reduce LA pressure. The prospective, non-randomized, open-label study, called “*A Transcatheter Intracardiac Shunt Device for Heart Failure with Preserved Ejection Fraction* “REDUCE LAP-HF”^31^ evaluated the performance and safety of this device in 64 patients with HFpEF and elevated PCWP. Preliminary analyses demonstrated clinical and hemodynamic benefits at 6 months. Pressure reductions in LA resulted in improved functional capacity, at the expense of a slight increase in the right cardiac pressure and output. These benefits persisted in a long-term evaluation with sustained improvement of the hemodynamic profile, NYHA functional class, quality of life and exercise capacity at the end of one year, with no evidence of complications.^32^

Subsequently, a randomized controlled phase II trial was performed with PCWP evaluation during exercise, one month after the implantation of the interatrial septal shunt device *vs*. sham procedure. It showed to be safe and effective, with a reduction of PCWP and without a significant increase in pulmonary artery pressure (PAP) or pulmonary vascular resistance, which are possible consequences of right cardiac overload.^33^ It remains unclear whether this hemodynamic effect will lead to sustained clinical improvements.

#### 13. Monitoring

Congestive symptoms are present in the majority of patients hospitalized for decompensated HF regardless of LVEF. Changes in body volume and cardiac filling pressures are predictive of adverse events. A strategy of hemodynamic monitoring, with consequent targeted and early therapeutic intervention, may reduce the risk of hospitalization for HF. The*Wireless pulmonary artery haemodynamic monitoring in chronic heart failure*, the “CHAMPION” trial,^34^ tested this hypothesis by using a microelectromechanical system pressure sensor permanently implanted during right cardiac catheterization. Through daily assessment of PAP and active reduction of filling pressures with diuretics and vasodilators, significant reductions were demonstrated in hospital admissions. The benefits persisted in the subgroup of patients with HFpEF, with reductions of 50% in HF hospitalizations after 17 months.^35^

#### 14. Exercise

Physical exercise is beneficial in certain conditions strictly related to HFpEF, such as hypertension and metabolic syndrome. The effect of structured and supervised training on exercise capacity, diastolic function and quality of life was evaluated. The *Exercise Training Improves Exercise Capacity and Diastolic Function in Patients With Heart Failure With Preserved Ejection Fraction, the “*EX DHF” trial,^36^ showed that a short-term supervised endurance/resistance training is achievable, safe, and effective in patients with HFpEF. The program maintenance in the long term and the involvement of elderly patients, at advanced stages of the disease and with multiple comorbidities, are possible limitations. Nevertheless, it seems a promising strategy with potential synergism with other pharmacological and non-pharmacological approaches. It is important to define the regimen approach, improve long-term adherence and expand availability.^37^

#### 15. Comorbidities

Another of the proposed pathophysiological mechanisms involves the existence of a systemic proinflammatory state induced by multiple comorbidities, resulting in endothelial dysfunction, cardiac remodeling and dysfunction. It was hypothesized that by screening and treating comorbidities in a targeted manner, the overall prognosis of these patients could be improved. The *Optimizing the Management of Heart Failure with Preserved Ejection Fraction in the Elderly by Targeting Comorbidities,* the “OPTIMIZE-HFpEF” trial,^38^ proposes a systematic screening and optimized treatment of comorbidities as a pathophysiological mechanism of HF, rather than the simple treatment of previously diagnosed concomitant pathologies. Although it lacks sufficient power to assess cost-effectiveness, it is a good starting point to test a new promising approach.

#### 16. Pacing

Patients with HFpEF and chronotropic incompetence may benefit from pacemaker devices, which may help to restore the normal HR during daily activity and exercise. The *Rate-Adaptive Atrial Pacing In Diastolic Heart Failure* (RAPID-HF) trial^39^ aims to evaluate the impact of this intervention on short-term exercise capacity, symptoms and quality of life.

#### 17. Iron Supplementation

Iron kinetics is part of the initial evaluation of patients with HF. Intravenous iron supplementation is part of the therapeutic approach in patients with HFrEF and reduced iron stores.^3^ The *Effect of IV Iron Ferric Carboxymaltose (Ferinject) on Exercise Tolerance, Symptoms and Quality of Life in Patients With Heart Failure With Preserved Ejection Fraction and Iron Deficiency With and Without Anaemia, the* “FAIR” trial,^40^ aims to evaluate the effect of intravenous iron on exercise capacity, quality of life, NYHA functional class, mortality and hospitalizations for HF in patients with HFpEF and iron deficiency, with or without anemia.

#### 18. Sodium-glucose cotransporter-2 inhibitors (SGLT2i)

HF and diabetes frequently coexist, associated with an increased risk of cardiovascular mortality and HF hospitalization.^41^ Several studies with SGLT2 inhibitors have demonstrated a significant reduction in HF hospitalizations in diabetic patients at high cardiovascular risk or with established cardiovascular disease (*Empagliflozin, Cardiovascular Outcomes, and Mortality in Type 2 Diabetes “EMPA-REG”,*^42^ *Canagliflozin and Cardiovascular and Renal Events in Type 2 Diabetes “CANVAS”,*^43^ *Dapagliflozin and Cardiovascular Outcomes in Type 2 Diabetes “DECLARE”*^44^ trials*).* Given the potential benefits of this pharmacological group in improving diastolic function in patients with HF,^45^ studies are underway to determine the impact of these drugs in patients with HFpEF, with and without diabetes (*A Phase III Randomised, Double-blind Trial to Evaluate the Effect of 12 Weeks Treatment of Once Daily EMPagliflozin 10 mg Compared With Placebo on ExeRcise Ability and Heart Failure Symptoms, In Patients With Chronic HeArt FaiLure With Preserved Ejection Fraction (HFpEF) “EMPERIAL - Preserved”,(*^46^*) Dapagliflozin in PRESERVED Ejection Fraction Heart Failure “PRESERVED-HF”,(*^47^*) EMPagliflozin outcomE tRial in Patients With chrOnic heaRt Failure With Preserved Ejection Fraction “EMPEROR-Preserved”(* ^48^*).*

## Conclusions

HFpEF is a common pathology, still poorly understood and without any treatment proven to be effective in reducing morbidity or mortality.

There seems to be no single cause to justify the failure of the obtained results; however, potential contributions can be identified: incomplete understanding of the pathophysiology, heterogeneity of the studied population, lack of universal diagnostic criteria with recruitment of patients without true HFpEF or at the very early stages, treatment not targeting the predominant pathophysiological mechanism, suboptimal designs or weak statistical power of the trials.

The pathophysiology of HFpEF is multifactorial, with several mechanisms and comorbidities involved, and probably different from those of HFrEF. It results from a complex interaction of factors that culminate in the reduction of cardiac and vascular functional reserve - systolic and diastolic dysfunction, atrial reserve, heart rate and rhythm, autonomic control, vasculature and microcirculation. The interaction and relative dominance of these factors make this pathology extremely heterogeneous. The definition and division into subgroups with certain phenotypes may allow a more targeted treatment, with possible improvement of the clinical results.

Several clinical trials are being carried out, using different therapeutic approaches. It is important to remember that these patients tend to be older and have multiple pathologies. Thus, the benefit of the treatments may be better evaluated by their effect on hospitalizations, functional status, symptoms and quality of life.
